# Urethral cavernous hemangioma: a highly misdiagnosed disease (a case report of two patients and literature review)

**DOI:** 10.1186/s12894-019-0441-0

**Published:** 2019-01-31

**Authors:** Fang Yong, Lin Juan, Wei Jinhuan, Yao Haohua, Chen Wei, Mo Jiacong, Luo Junhang, Wang Wenwei

**Affiliations:** 1 Department of Urology, First Affiliated Hospital of Sun Yat-sen university, Guangzhou, 510080 China; 2 Department of Pediatrics, Third Affiliated Hospital of Sun Yat-sen university, Guangzhou, 510630 China

**Keywords:** Urethra cavernous hemangioma, Urethremorrhage, Pingyangmycin

## Abstract

**Background:**

Diagnosis of urethral cavernous hemangioma (UCH) is very rare. It can be easy to misdiagnose and mistreat due to its atypical clinical manifestations and a lack of relevant knowledge. The study is to explore the diagnosis, differential diagnosis, and treatment of UCH.

**Case presentation:**

The first patient was a 15-year-old male, who was admitted to the hospital for more than 1 year with repeated hematuria. UCH was diagnosed by cystoscope biopsy, and cured with local injection of pingyangmycin. The second patient was a 49-year-old male, who was admitted for repeated painless gross hematuria and intermittent urethral bleeding after penile erection for more than 20 years. The case had been misdiagnosed as seminal vesiculitis, urethritis, or prostatitis, for over 20 years, until it was diagnosed as UCH by MR examination of the penis. It was treated by injection of pingyangmycin into the hemangioma’s lumen and base. A small incision in the ventral penile area was separated from the location of the hemangioma, which was injected with pingyangmycin again. A biopsy of resected tissue further confirmed the diagnosis of UCH.

**Conclusions:**

UCH is an easily misdiagnosed disease. Intermittent painless hematuria is important characteristic of UCH. Local injection of pingyangmycin is a good option for treatment of UCH.

**Electronic supplementary material:**

The online version of this article (10.1186/s12894-019-0441-0) contains supplementary material, which is available to authorized users.

## Background

Urethral cavernous hemangioma (UCH) is an unusual disease and a few cases have been reported [1–7]. It can be easy to misdiagnose and mistreat UCH due to its atypical clinical manifestations and a lack of relevant knowledge. Two patients were admitted in our hospital in November 2002 and April 2013 respectively that were misdiagnosed during the preliminary diagnoses. The diagnoses were corrected by cystourethroscopy or imaging. The clinical characteristics and treatment methods of these cases are summarized here.

## Case presentation

**Fig. 1:**
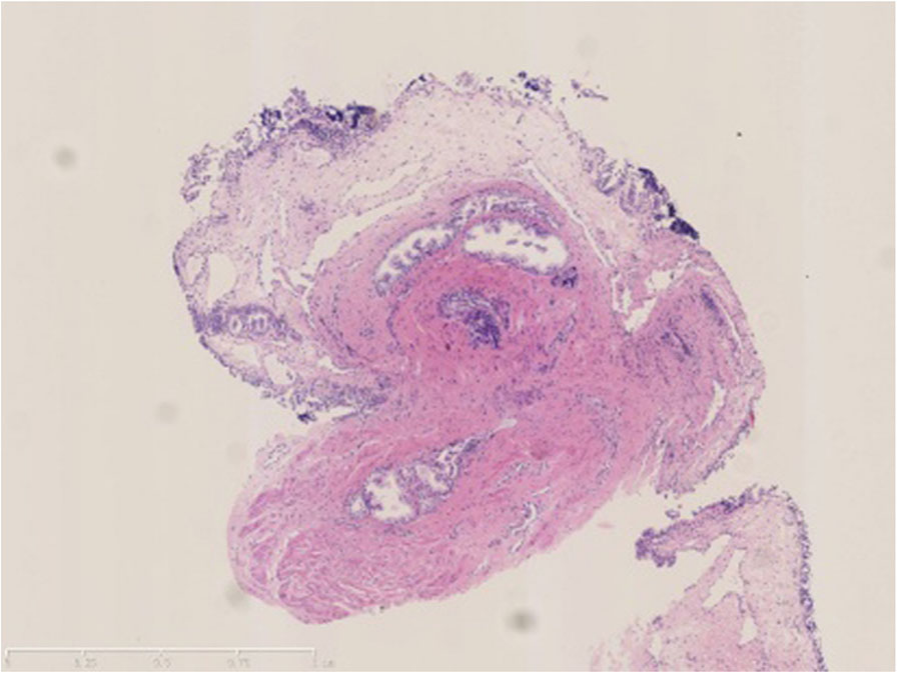
The pathological tissue originates from the urethral mucosa, displaying submucosal vascular dilatation of the urethra, which is consistent with urethra cavernous hemangioma (UCH)

The first patient was a 15-year-old male. After 1 year of repeated gross hematuria, he was admitted to the Department of Nephrology in our hospital on November, 2002. Urinary system ultrasound, intravenous pyelography, contrast enhancement and plain CT scans of the kidney, and renal biopsy were performed. However a cause of the patient’s hematuria could not be identified. Later, the patient was transferred to pediatric surgery and cystourethroscopy was performed. The results showed urethral mucosa edema, mass and miliary bulging, and bleeding of the membranous urethra. The urethral mucosa biopsy was also performed, and the pathological report displayed submucosal vascular dilatation of the urethra which is consistent with UCH (Fig. 1). Two weeks after the cystourethroscopy, pingyangmycin was injected under the cystoscope in the outpatient department of urology. In the operation, 4 mg of pingyangmycin was injected into the bulge on the urethral membrane. The urethral catheter was retained and removed after 3 days. At the follow-ups 1 year, 12 years, and 15 years after treatment, gross hematuria did not recur, and micturition and erectile function were normal.

**Fig. 2:**
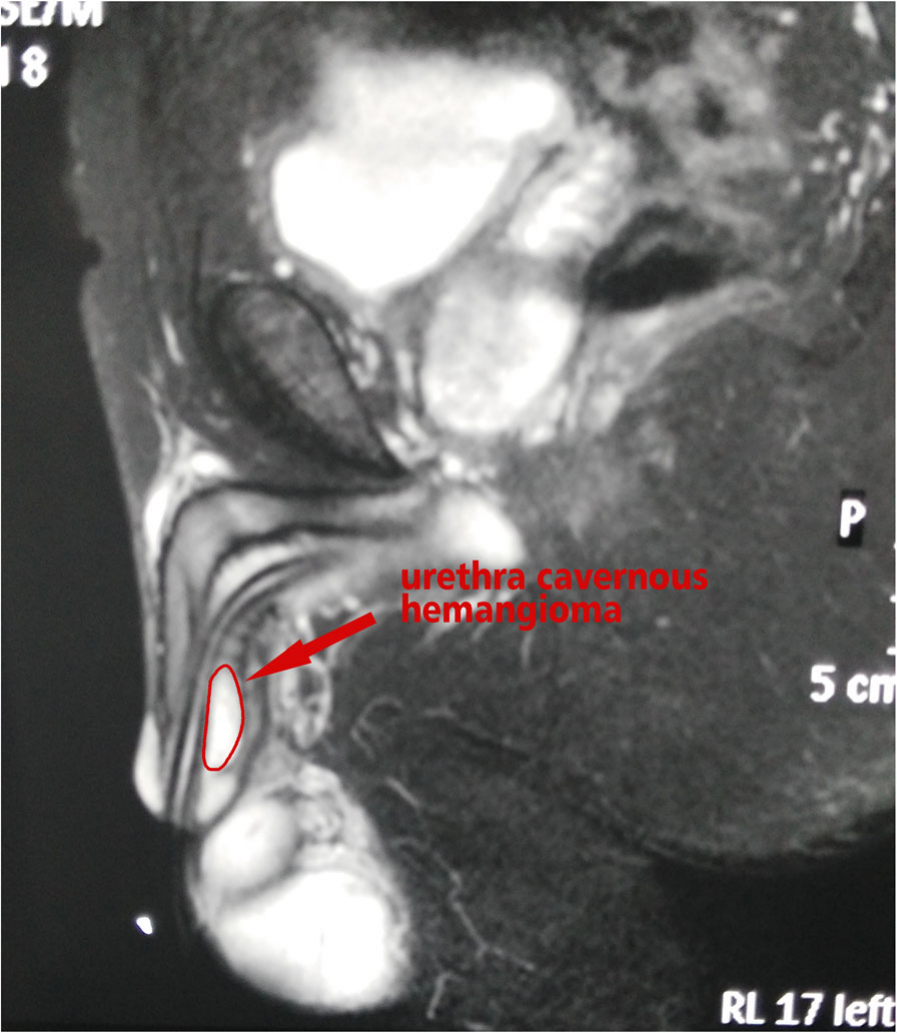
Preoperative penis sagittal MRI shows a high signal on the urethra cavernous body at the front of the penis. The range was about 1.1 × 2.4 cm

**Fig. 3:**
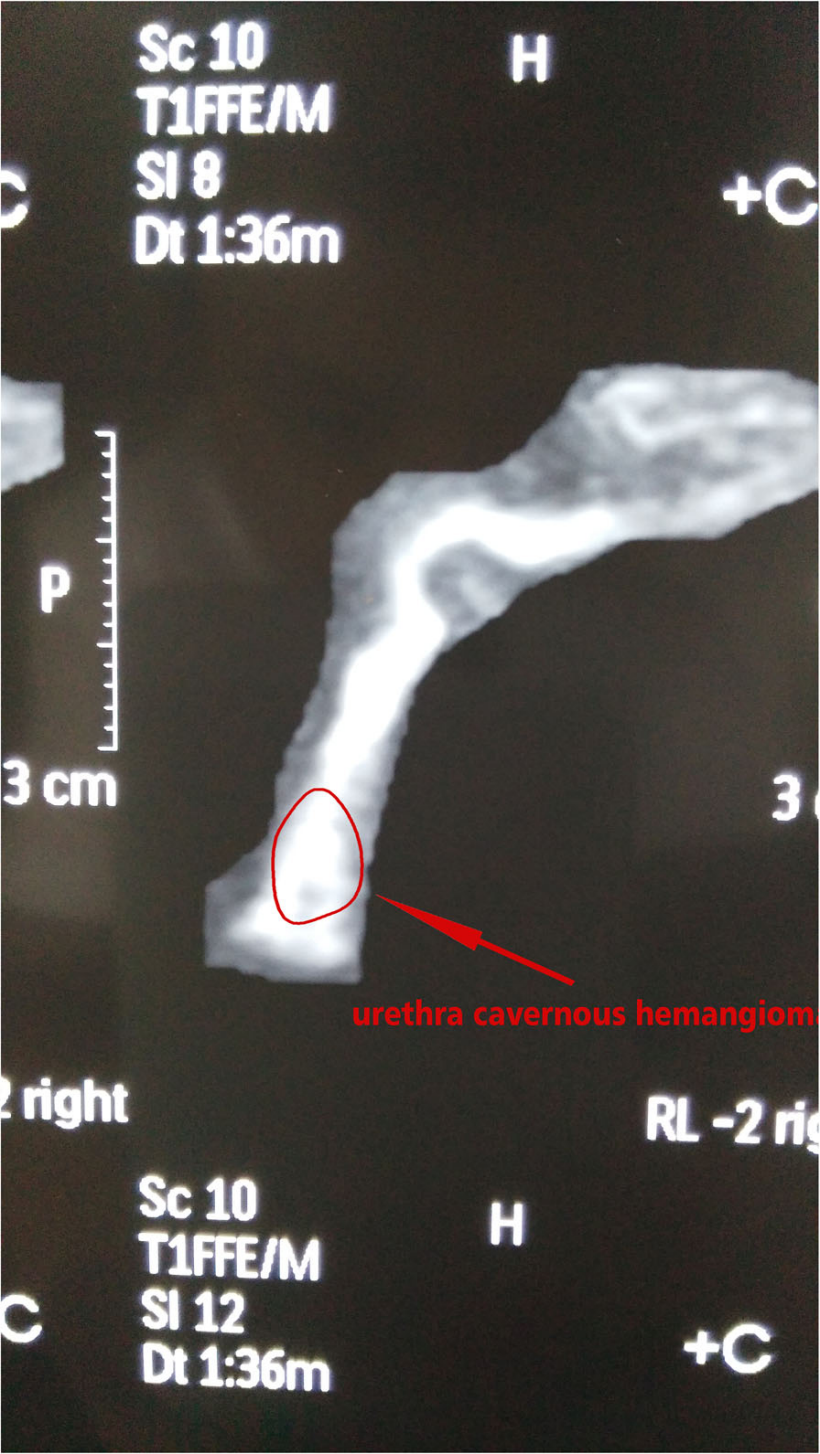
Preoperative penis MRI reconstruction shows a high signal at the front of the penis

**Fig. 4:**
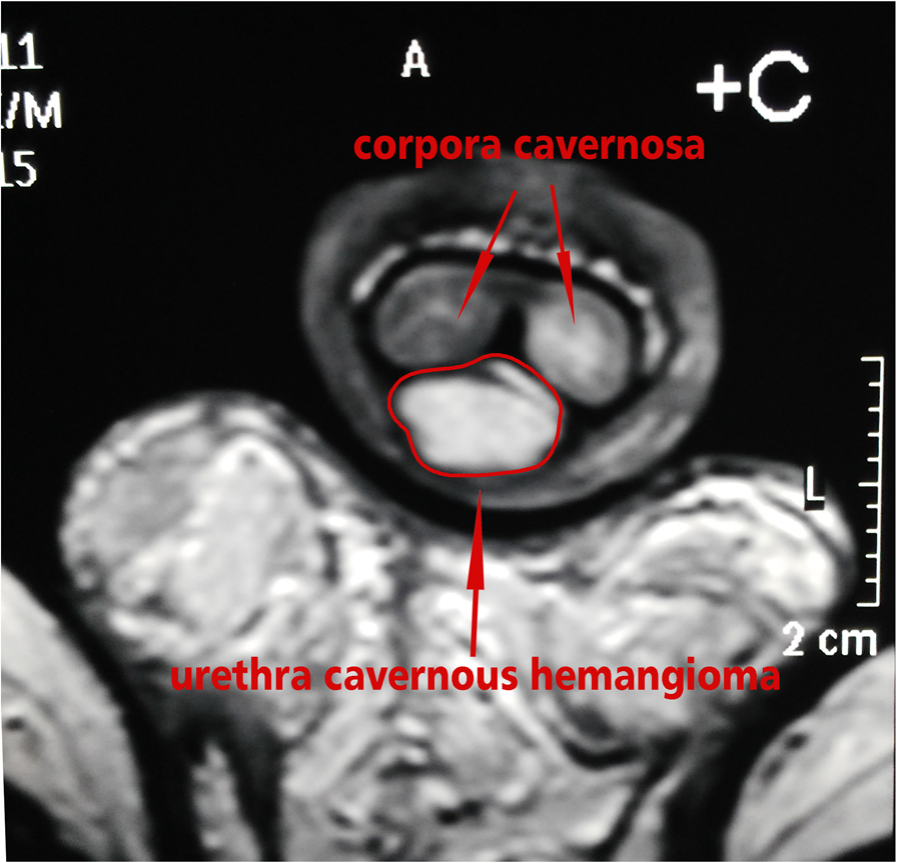
Preoperative penis coronal MRI shows a high signal on the ventral side of the penis, and underneath the urethra cavernous bodies of the penis

The second patient was a 49-year-old male with repeated painless gross hematuria and discontinuous urethral bleeding after penile erection for more than 20 years, which had been aggravated for 4 months. He was admitted to the Department of Urology of our hospital on April 29, 2013. The patient had also been misdiagnosed in a local hospital over the course of 20 years with seminal vesitis, urethritis, or prostatitis. No obvious improvement was observed with treatment. Cystoscopy performed in local hospitals, revealed no obvious abnormalities. After artificial erection by tightening the root of the penis and injecting saline into the corpus cavernosum, a small amount of bloody liquid could be detected in the urethra. The penis MR showed an abnormal signal on the right side of the urethra cavernous body at the front of the penis. The range was about 1.1 × 2.4 cm. The distal portion closed to the urethral meatus. The proximal portion was at a distance of 2.4 cm from urethral meatus and invaded the right side of the glans (Fig. 2, 3 and 4). After artificial erection of the penis, urethroscopy examination showed that there was a 0.3 cm fissure located in the 11 o’clock urethral mucosa 2 cm away from the urethral meatus. The fissure bled and the bleeding was aggravated when the penis was squeezed (Additional file 1: Video S1). Pingyangmycin was injected into the lumen and basal side of the tumor under the urethroscope. We took a biopsy from the small incision on the ventral side of the penis that separated the hemangioma and continued to inject pingyangmycin. The total dose of pingyangmycin was 8 mg. The catheter inserted after injection was removed 3 days after the operation. The postoperative pathological report reported a diagnosis of UCH. At 1 year and 5 years of follow-ups, no bleeding occurred during or after penile erection, no gross hematuria recurred, and penile erection and voiding were normal.

## Discussion and conclusions

As the tissue of the urethral cavernous body is half-wrapped around the glans and the cavernous body of the penis, along with in the relative depth of the penis, the growth of the hemangioma is limited. Therefore, UCH often has no obvious signs. However, if it breaks through to the urethra it becomes noticeable, making gross hematuria the most important clinical feature of the diseases.

UCH can occur at any age in males, and is more likely to occur in young people. Local trauma and urinary tract inflammation are often the main factors of tumor rupture and bleeding. Penile erection in adult males leads to vascular dilatation and venous flow obstruction, which can also cause rupture and bleeding of UCH. Blockage of the urethra can cause dysuria, in which a larger UCH can form or bleeding can result in a blood clot [1]. In sum, intermittent painless gross hematuria and gross hematuria are the most important features of this disease.

However, the atypical features of UCH lead the disease being easily misdiagnosed and mistreated for a long time. In this report, the first patient was misdiagnosed as nephritis, which was removed from the diagnosis until the invasive renal biopsy was performed. The second patient was misdiagnosed and mistreated for more than 20 years. Therefore, for the painless hematuria that has been excluded from the urinary system related diseases such as inflammation, malignant tumor and stone, especially for young people, cystoscopy is an important means of examination.

Another important feature is that hematuria is often aggravated after penile erection, especially for the sexually active adult male, even visible blood dripping from the urethral meatus. This is very similar to the characteristics of seminal vesicle inflammation and is also one of the reasons for misdiagnosis. In the second patient, as a 49-year-old adult male patient, the most frequent misdiagnosis was seminal vesicle inflammation. The reason why we could correct the misdiagnosis is we noted that the urethral bleeding after the patient’s sexual activity was not discharged with the semen. Therefore, for incurable hemospermia of patients’ complaining, we should pay more attention to the way that the blood is discharged from the urethra and the relationship with the excretion of sperm so as to distinguish between the semen with blood (the so-called true hemospermia) or the urethral bleeding caused by sexual activity.

At present, the common treatment of hemangioma includes drugs, physical therapy, and surgery. Drug treatments include oral propranolol, oral or injection of glucocorticoid, local injection of pingyangmycin, etc. [8, 9]. Physical therapies include direct current copper needle, laser, radiation, microwave, etc. [10–13]. Surgery treatment includes local excision, the purse-string closure method, etc. [14]. In particular, minimally invasive urological surgery like transurethral Holmium laser therapy is also suitable for the treatment of hemangioma of the urinary system, which has been successfully applied to urethral hemangioma [15]. The surgical excision of UCH is a complex operation with significant trauma because of the deep location of the mass and proximity to the urethra. Urethral stricture may be caused by scarring after the operation or the surgical injury leads to acquired hypospadias. Radiation therapy also causes radiation orchitis, which may affect reproductive function [16].Therefore, both patients were treated with the interventional technology pingyangmycin injection, and satisfactory results were achieved.

Pingyangmycin is a commonly used antitumor drug. This drug mainly inhibits the synthesis of deoxynucleic acid and ribonucleic acid, decomposes deoxyribonucleic acid, breaks the DNA chain and produces oxygen free radicals, prevents DNA replication, and interferes with cell division and proliferation [17]. Hemangioma is caused by abnormal proliferation of vascular endothelial cells. Injection of pingyangmycin in the tumor, can quickly inhibit the proliferation of immature vascular endothelial cells. At the same time, its chemical stimulation can cause aseptic inflammation of vascular endothelium and destroy the endothelium of the blood sinus, resulting in damage of the vascular endothelium and extensive formation of microthrombus, causing local tissue degeneration. Necrosis and coagulation eventually leads to tissue fibrosis, bringing about the gradual disappearance of hemangioma [18]. Further mechanistic studies have shown that pingyangmycin can induce apoptosis and reduce cell invasiveness by inhibiting the PI3K/Akt pathway [19] and by up-regulating P53 inducing P53-dependent apoptosis [20].

Our study is limited because it included only two patients from China. And no female patients included. Our application of pingyangmycin injection for treatment of UCH may be optimal only to patients with small tumor size.

In conclusion, UCH is a hemangioma deep in the penis. The most common presentation of UCH is intermittent hematuria which is typically painless. This disease is easily misdiagnosed. For patients complaining with long lasting hematuria or bloody sperm, we should inquire about the characteristics of urinating blood and its relationship with the discharge of sperm. For suspected patients, we should take a cystoscopy and MR of the penis. It is advisable to apply pingyangmycin injection for treatment, and the prognosis is generally good.

## Additional file

Additional file 1: **Video S1.** Urethroscopy examination video. After artificial erection of the penis, urethroscopy showed that the 0.3 cm fissure located in the 11 o’clock urethral mucosa 2 cm away from the urethral meatus. The fissure bled and the bleeding was aggravated when the penis was squeezed. (AVI 2906 kb)

## Abbreviations

MR: Magnetic resonance
UCH: Urethra cavernous hemangioma
